# Using a Stacked Autoencoder for Mobility and Fall Risk Assessment *via* Time–Frequency Representations of the Timed Up and Go Test

**DOI:** 10.3389/fphys.2021.668350

**Published:** 2021-05-28

**Authors:** Shih-Hai Chen, Chia-Hsuan Lee, Bernard C. Jiang, Tien-Lung Sun

**Affiliations:** ^1^Department of Industrial Engineering and Management, Yuan Ze University, Taoyuan, Taiwan; ^2^Department of Industrial Management, National Taiwan University of Science and Technology, Taipei, Taiwan

**Keywords:** SAE, TFA, DNNs, wavelet transform, LDA

## Abstract

Fall risk assessment is very important for the graying societies of developed countries. A major contributor to the fall risk of the elderly is mobility impairment. Timely detection of the fall risk can facilitate early intervention to avoid preventable falls. However, continuous fall risk monitoring requires extensive healthcare and clinical resources. Our objective is to develop a method suitable for remote and long-term health monitoring of the elderly for mobility impairment and fall risk without the need for an expert. We employed time–frequency analysis (TFA) and a stacked autoencoder (SAE), which is a deep neural network (DNN)-based learning algorithm, to assess the mobility and fall risk of the elderly according to the criteria of the timed up and go test (TUG). The time series signal of the triaxial accelerometer can be transformed by TFA to obtain richer image information. On the basis of the TUG criteria, the semi-supervised SAE model was able to achieve high predictive accuracies of 89.1, 93.4, and 94.1% for the vertical, mediolateral and anteroposterior axes, respectively. We believe that deep learning can be used to analyze triaxial acceleration data, and our work demonstrates its applicability to assessing the mobility and fall risk of the elderly.

## Introduction

Remote health monitoring has been gaining increased interest as a way to improve the quality and reduce the costs of healthcare, especially for the elderly (Seyfioğlu et al., 2017). According to the World Health Organization, a person aged 65 years and over has a fall risk of 28–35%, which increases to 32–42% for those aged over 70 years [World Health Organization [WHO], 2007]. According Letts et al. (2010), 33% of community-dwelling elderly have experienced a fall event, and 50% fall repeatedly. About one-third of elderly people fall every year, and the chance of falling increases with age [World Health Organization [WHO], 2007; Bergland, 2012]. Falling can have serious long-term consequences for the elderly, including hospitalization, decreased mobility, fear of falling and even death. Older people with gait, mobility or balance problems are at higher risk of falling in the future (Ganz et al., 2007; Cuevas-Trisan, 2017). To develop an effective fall prevention program, elderly people with a fall risk must first be identified.

Various factors drive the fall risk. Mitchell et al. (2012) showed sarcopenia, the typical age-related decline in skeletal muscle mass cause strength reduction as well as balance issue. Poor balance and mobility have been validated as a key cause of falls among the elderly. Continuous monitoring could be a practical approach to reduce and prevent falls by providing early warnings to facilitate appropriate interventions (Shany et al., 2012). However, continuous monitoring of gait and postural stability requires extensive healthcare and clinical resources. Limited professional resources (e.g., physical therapists, nurses, and doctors) are insufficient for detecting balance deterioration in a timely fashion, especially as the aged population increases worldwide. This can result in many falls that could have been avoided through continuous monitoring and early intervention. To fill the gap between available resources and care needs, an approach is needed for assessing the balance and mobility of the elderly in a timely manner without involving healthcare professionals.

Wearable systems based on inertial sensors are light, portable, and cheap, and they can be used to quantify body motions. Previous research (Howcroft et al., 2013) on fall risk assessment focused on feature-based methods, in which many related features are derived with domain knowledge. This requires multiple feature engineering steps before the classification or discrimination results can be obtained. The timed up and go test (TUG) is commonly used to evaluate mobility and the fall risk of the elderly in hospital and community environments (Podsiadlo and Richardson, 1991; Barry et al., 2014). Tri-axial acceleration sensors can be used to obtain time-domain signals during TUG (Wu et al., 2019; Lee et al., 2020), which can be transformed through time–frequency analysis (TFA) to extract time-domain, frequency-domain, and spectral energy-related information. Since the past literature (Cardozo et al., 2011) and (Garcia-Retortillo et al., 2020) has shown investigating spectral power distribution of muscle (using accelerometer data or related physiological parameters, such as EMG) and its response to fatigue and aging in elderly subjects, we can use spectral energy-related information to assess fall risk of elderly subjects *via* TUG test.

Nweke et al. (2018) showed that deep neural network (DNN) methods are being adopted for automatic feature learning in diverse fields such as health, image classification, and recently, for the feature extraction and classification of simple and complex human activity recognition in mobile and wearable sensors. They also provided further insights on deep learning based on the decision fusion of human activity recognition for enhanced performance accuracy. Hossain et al. (2018) showed that deep learning architectures have been increasingly used in activity recognition problems that empower several application domains that require considerably less human supervision in the process. Moreover, they showed that such architectures are gaining increasing popularity for extracting meaningful information from these large volumes of data. DNNs are suitable for TFA owing to their excellent discrimination of images. The non-stationary nature of the TUG signal indicates that TFA can be used for motion identification in general and fall detection in particular (Jokanovic et al., 2016a, July). Deep learning can be used to capture the detailed and complex properties of the TF signature and feed the learned underlying features to the classifier (Jokanovic et al., 2016a, May). An autoencoder (AE) is a feed-forward neural network that aims to reconstruct the input at the output under certain constraints. Seyfioğlu et al. (2018) proposed an unsupervised pre-training algorithm for initializing the AE weights and bias that is highly effective when only a small number of labeled training samples are available. The stacked autoencoder (SAE) is a DNN that can classify highly similar classes of aided and unaided walking, as might be encountered in assisted-living environments for the elderly, and it has been applied in recognizing 12 different gaits (Seyfioğlu et al., 2017) as well as in fall detection.

In this paper, we propose the use of sensor and DNN-based technology, apply TFA to convert tri-axial accelerometer data and deep learning-based latent feature representation with a SAE to develop a surrogate approach for assessing the mobility function and fall risk detection of the elderly. And DNN-based analysis techniques will be an available approach for continuous monitoring in the future.

## Materials and Methods

We considered two evaluation methods for fall risk: feature-based and DNN-based evaluation. Feature-based evaluation, based on traditional statistical features and method for evaluation, combines feature extraction, feature selection and classifier, and it relies on heuristic handcrafted feature design. By contrast, DNN-based evaluation in this paper is based on the SAE and a softmax classifier layer, and it can automatically learn better feature representations than the handcrafted ones (Ng., 2011). Leave-one-out cross-validation was employed for both evaluation methods to ensure a robust classification accuracy. The results of the two evaluation methods were then compared.

### Subjects

Our study took place at a hospital in central Taiwan between April 2014 and May 2015. We recruited and selected 44 elderly subjects dwelling in a community. A medical professional team that included rehabilitation physicians, physiotherapists and functional therapists performed TUG to evaluate the mobility function of the subjects. Prior to the evaluation, written consent was obtained from the subjects. The subjects were over 60 years of age, had no history of musculoskeletal injuries or central nervous system problems in the last 3 months and could walk independently without any help. Valid data were obtained for 44 elderly subjects with a mean age of 78.18 ± 7.97 years. There were 14 male subjects with an average age of 80.43 ± 5.60 years and 30 female subjects with an average age of 77.13 ± 8.74 years.

### Sensor

**FIGURE 1:**
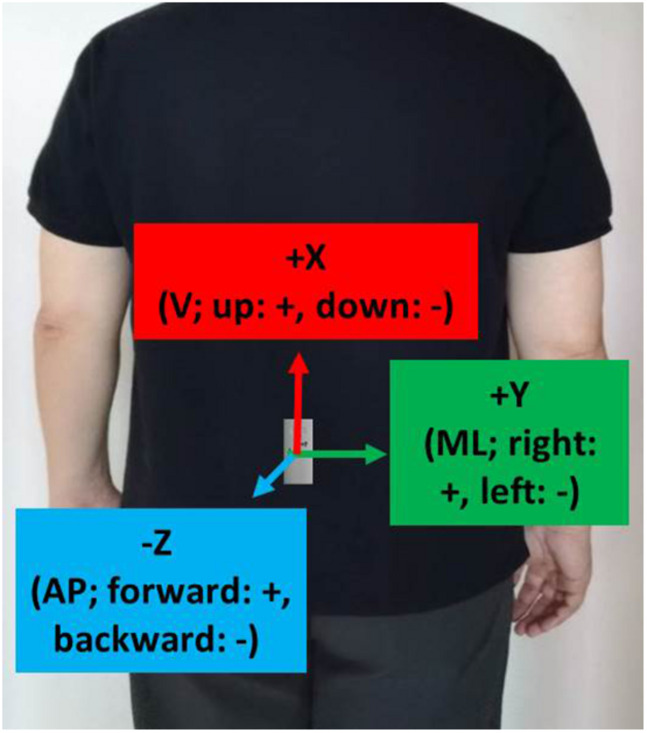
Sensor locations and corresponding axes/directions.

As shown in Figure 1, a tri-axial accelerometer (RD3152MMA7260Q, Freescale Semiconductor-NXP, United States) with a sampling rate of 45 Hz was placed at vertebrae L3–L5 on a subject’s back for the TUG experiments. L3–L5 correspond to the center of gravity of the human body and are used in most fall risk assessments (Howcroft et al., 2013). The X-, Y-, and Z-axes were aligned with the vertical (V; up: +, down: −), mediolateral (ML; right: +, left: −), and anteroposterior (AP; forward: +, backward: −) directions, respectively.

### Timed Up and Go Test

**FIGURE 2:**
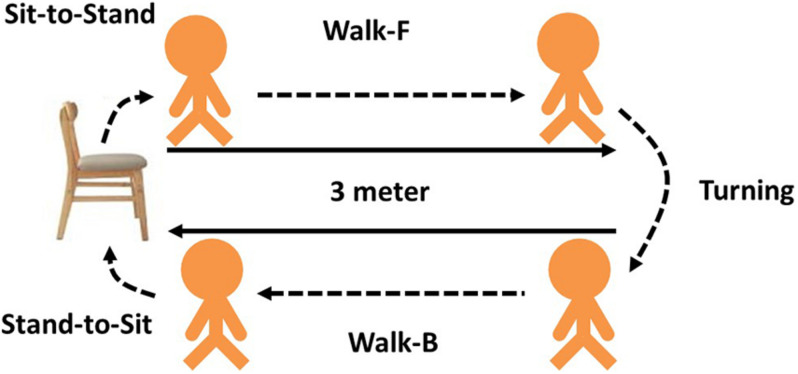
Five phases of TUG.

Each subject was asked to perform a TUG. The observer marked the start and end times. As shown in Figure 2, each TUG was divided into five phases or subtasks: from sitting to standing (sit-to-stand), walking forward (walk-F), reaching the 3-m mark and turning around (turning), walking backward (walk-B) and reaching the chair and returning to sitting (stand-to-sit). The TUG time was recorded, and a threshold time was determined to classify subjects as a fall risk or not a fall risk. Alexandre et al. (2012) recommend that it is considered a high fall risk if the time of community elderly for TUG is greater than 12.47 s.

### Feature-Based Evaluation

For feature-based evaluation, the features of the axial signals were obtained by referring to past literature (Banos et al., 2014). The most widely used features include the mean, standard deviation, maximum, minimum, and mean crossing rate (MCR). The mean and standard deviation are used to express the average and variation of the force for each axial signal. The maximum and minimum express the largest and smallest values of the signal for the entire domain. The MCR is the rate at which data cross the average value, and it has been widely used in signal recognition and physical activity recognition (Gao et al., 2014; Arivu et al., 2018; Bountourakis et al., 2019).

Features were selected for the feature-based evaluation according to their significance (Wu et al., 2019; Lee et al., 2020). The significance was obtained through Student’s *t*-test. A feature was considered significant if *p* ≤ 0.05. In addition, linear discriminant analysis (LDA) was performed to obtain a confusion matrix for evaluating the performance.

### Deep Neural Network-Based Evaluation

**FIGURE 3:**
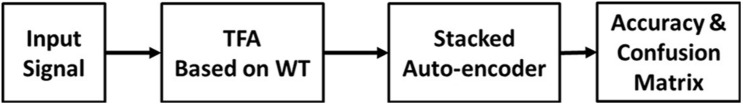
Flowchart for DNN-based evaluation.

Figure 3 shows the flowchart of the DNN-based evaluation. The input signal was the tri-axial data collected during the TUG experiments. TFA was applied to the data, and the SAE was applied in classifying the signal. Finally, the accuracy and confusion matrix were obtained.

#### Time–Frequency Analysis

A time–frequency representation (TFR) is a view of a signal, which is taken as a function of time, in both time and frequency domains. TFA can be applied to a time series signal to observe the time-domain, frequency-domain and spectral-energy information simultaneously. TFA based on wavelet transform (WT) is widely used in biomedical science for applications such as fall detection (Jokanovic et al., 2016a, July; Jokanovic et al., 2016b, May) and analysis of electroencephalography (Yordanova et al., 2013) and electromyography (Zia ur Rehman et al., 2018). In this study, the Morlet wavelet was used for TFA of the tri-axial acceleration signal from the TUG experiments. This method was described in previous literature (Tallon-Baudry et al., 1997). The complex Morlet wavelet _*w(t,f_c_)*_ can be generated in the time-domain for different frequencies *f* as follows:

(1) $$w\,\left(t,f_{c}\right)=A\,\exp\,\left(-t^{2}/2\,\sigma_{t}^{2}\right)\,\exp\,\left(i\,2\,\pi \,f_{c}\,t\right),$$

where *t* is the time, σ_*t*_ is the wavelet duration, normalization factor $A={\left(\sigma_{t}\,\sqrt{\pi }\right)}^{-1/2}$, a constant ratio of*f_c_*/σ_*f*_ = 7 was used. *f*_*c*_ is the central frequency, and σ_*f*_ is the width of the Gaussian shape in the frequency-domain. For different *f*, the time and frequency resolutions can be calculated as 2σ_*t*_ and 2σ_*f*_, respectively, where σ_*t*_ = 1/2πσ_f_. Finally, the time-varying energy |*E*(*t*,*f*_*c*_)| of the signal [*s*(*t*)] is calculated by squaring the absolute value of the convolution of the signal with the complex Morlet wavelets:

(2) $$E\,\left(t,f_{c}\right)={\left|w\,\left(t,f_{c}\right)\times s\,\left(t\right)\right|}^{2}.$$

In this study, the frequency range was swept from 0.05 to 5 Hz, and a TF image was obtained for classification by the SAE.

#### Stacked Autoencoder Network Architecture

A neural network with multiple hidden layers can be used to solve classification problems with complex data such as images. Each layer can learn features at a different level of abstraction. However, training a neural network with multiple hidden layers can be difficult. In this paper, we use the SAE structure, which is a DNN based on the AE concept. An AE is a neural network comprising an encoder, followed by a decoder, and it attempts to replicate its input at its output. We used an AE so that the hidden layers can be trained individually in an unsupervised fashion. No labeled data are required for training or learning. The encoder maps the input *x* to a new representation *z*, which is decoded back at the output to reconstruct the input $\hat{x}$: (Hinton and Salakhutdinov, 2006; Zia ur Rehman et al., 2018; MATLAB autoencoder, 2021).

(3) $$z=h_{1}\,\left(W_{1}\,x+b_{1}\right),$$

(4) $$\hat{x}=h_{2}\,\left(W_{2}\,z+b_{2}\right),$$

where _*h* _1_ and _*h* _2_ are activation functions, _*W* _1_ and _*W* _2_ are weight matrices and _*b* _1_ and _*b* _2_ are bias vectors for the encoder and decoder, respectively. Each layer can learn features with a different level of abstraction. If the number of hidden neurons is less than the number of input neurons, then the AE attempts to learn a sparse representation of the input data (Jokanovic et al., 2016a, July). Sparsity can be encouraged for an AE by adding a regulariser to the cost to prevent overfitting (Zia ur Rehman et al., 2018). In this study, the input was a color image with a resolution of 28 × 28 pixels and three channels (28 × 28 × 3 = 2,352 pixels). The AE had two hidden layers. The logistic sigmoid was used for both layers in the encoder and decoder.

In an SAE, the output of one AE is fed to the input of another AE, and sparsity is encouraged by adding regularization to the cost for neuron *i*. The average output activation for neuron *i* can be formulated as (MATLAB autoencoder, 2021):

(5) $${\hat{p}}_{i}=\frac{1}{n}\,\sum_{j=1}^{n}z_{i}\,\left(x_{j}\right),$$

where *i* is the *i*th neuron, *n* is the total number of training examples and *j* is the *j*th training example. A regulariser is introduced to the cost function using the Kullback–Leibler divergence: (Kullback, 1997; Zia ur Rehman et al., 2018).

(6) $${\text{Ω}}_{\text{sparsity}}=\sum_{i=1}^{d}p\,\log\,\left(\frac{p}{{\hat{p}}_{i}}\right)+\left(1-p\right)\,\log\,\left(\frac{1-p}{1-{\hat{p}}_{i}}\right),$$

where *d* is the total number of neurons in a layer and *p* is the desired activation value (i.e., sparsity proportion). The L2 regularization term _Ω _weights_ is also added to the cost function to control the weights:

(7) $${\text{Ω}}_{\text{weights}}=\frac{1}{2}\,\sum_{l}^{L}\sum_{j}^{N}\sum_{i}^{K}{\left(w_{\mathrm{ji}}^{l}\right)}^{2},$$

where *L* is the number of hidden layers, *N* is the total number of observations and *K* is the number of features within an observation.

By inserting the regularization terms from Eqs 6, 7 into the mean squared error of the reconstruction, the cost function can be formulated as follows:

(8) $$\text{E}=\frac{\frac{1}{N}\,\sum_{n=1}^{N}\sum_{k=1}^{K}{\left(x_{\mathrm{kn}}-{\hat{x}}_{\mathrm{kn}}\right)}^{2}}{\mathrm{mean}\,\mathrm{square}\,\mathrm{error}}+\lambda \cdot \frac{{\text{Ω}}_{\text{weights}}}{\begin{array}{c} \text{L}\,2\,\text{Regularization} \end{array}}+\beta \cdot \frac{{\text{Ω}}_{\text{sparsity}}}{\begin{array}{c} \text{Sparsity}\,\text{Regularization} \end{array}},$$

where λ is the coefficient for L2 regularization to prevent overfitting and β is the coefficient for sparsity regularization that controls the sparsity penalty term (MATLAB autoencoder, 2021).

Ju et al. (2015) and Coates et al. (2011) showed that the number of neurons in the hidden layer of a DNN may be more important than the feature-learning algorithm and model depth. In addition, the combinatorial space required to explore all possible combinations of hyperparameters is huge (Tsinalis et al., 2016). Therefore, we focused on locally optimizing the number of neurons for two layers and obtained the minimum mean squared error according to Eq. 8. The other parameters were taken from MATLAB: λ was set to 0.004 and 0.002 for the first and second hidden layers, respectively, β = 4 for both hidden layers and *p* was 0.015 and 0.01, respectively. After unsupervised training, the decoder was removed from the network, and the remaining encoder components were trained in a supervised manner by adding a softmax classifier with two neurons after the encoder. The softmax classifier is an advanced version of probability-based logistic regression and is often used in the final layer of a neural network. Finally, the SAE was obtained.

## Results and Discussion

Subjects were considered a fall risk if their TUG time was greater than 12.47 s and not a fall risk if the TUG time was less than 12.47 s. Table 1 lists the demographic data of the at-risk subjects (*n* = 22) and no-risk subjects (*n* = 22).

**TABLE 1:** Demographic data of subjects at-risk of falling and not at-risk.

	**Fall risk (*n* = 22)**	**Non-fall risk (*n* = 22)**
Age	78.18 ± 7.97	76.59 ± 9.16
Gender		
Male	8	6
Female	14	16

### Feature-Based Analysis of the Timed Up and Go Test Results

Table 2 details the *t*-test results for the significance of the 15 statistical features of the tri-axial acceleration data. Eight significant features were identified (Mean_V, Std_V, Max_V, MCR_V, Max_ML, MCR_ML, Max_AP, and MCR_AP), which are aligned with normality by using Kolmogorov-Smirnov test, and LDA was applied to each axis. Table 3 presents the classification results. The classification accuracies along the X-axis (V), Y-axis (ML), and Z-axis (AP) were 79.5, 81.8, and 75.0%, respectively. The sensitivities were 72.7, 81.8, and 72.7%, respectively. The specificities were 86.4, 81.8, and 77.3%, respectively. These results were then used for comparison to the DNN-based evaluation.

**TABLE 2:** Statistical features of TUG data for subjects.

**Statistic features**		**Fall risk (*n* = 22)**	**Non-fall risk (*n* = 22)**	***p*-value**
V-axis	Mean_V	0.949 ± 0.272	1.333 ± 0.308	0.000**
	Std_V	1.217 ± 0.360	1.709 ± 0.341	0.000**
	Max_V	4.063 ± 1.603	4.899 ± 1.061	0.049*
	Min_V	−3.504 ± 1.314	−4.122 ± 1.280	0.122
	MCR_V	0.098 ± 0.015	0.087 ± 0.010	0.008*
ML-axis	Mean_ML	0.994 ± 0.251	0.979 ± 0.205	0.826
	Std_ML	1.131 ± 0.297	1.247 ± 0.251	0.167
	Max_ML	2.846 ± 0.981	3.622 ± 1.241	0.027*
	Min_ML	−2.919 ± 0.833	−3.422 ± 0.959	0.07
	MCR_ML	0.060 ± 0.014	0.084 ± 0.018	0.000**
AP-axis	Mean_AP	2.075 ± 0.765	1.666 ± 0.597	0.055
	Std_AP	1.823 ± 0.382	1.892 ± 0.336	0.528
	Max_AP	1.352 ± 0.862	2.055 ± 0.834	0.009*
	Min_AP	−7.544 ± 1.352	−7.057 ± 1.494	0.263
	MCR_AP	0.050 ± 0.014	0.063 ± 0.014	0.005*

*Indicate *p* < 0.05 between two groups. **Indicate *p* < 0.005 between two groups.

**TABLE 3:** Classification results for LDA classifiers.

**Axis**	**Acc.**	**Sen.**	**Spec.**
V-axis	79.50%	72.70%	86.40%
ML-axis	81.80%	81.80%	81.80%
AP-axis	75.00%	72.70%	77.30%

### Deep Neural Network-Based Analysis of Timed Up and Go Test Results

#### Analysis of TF Images

**FIGURE 4:**
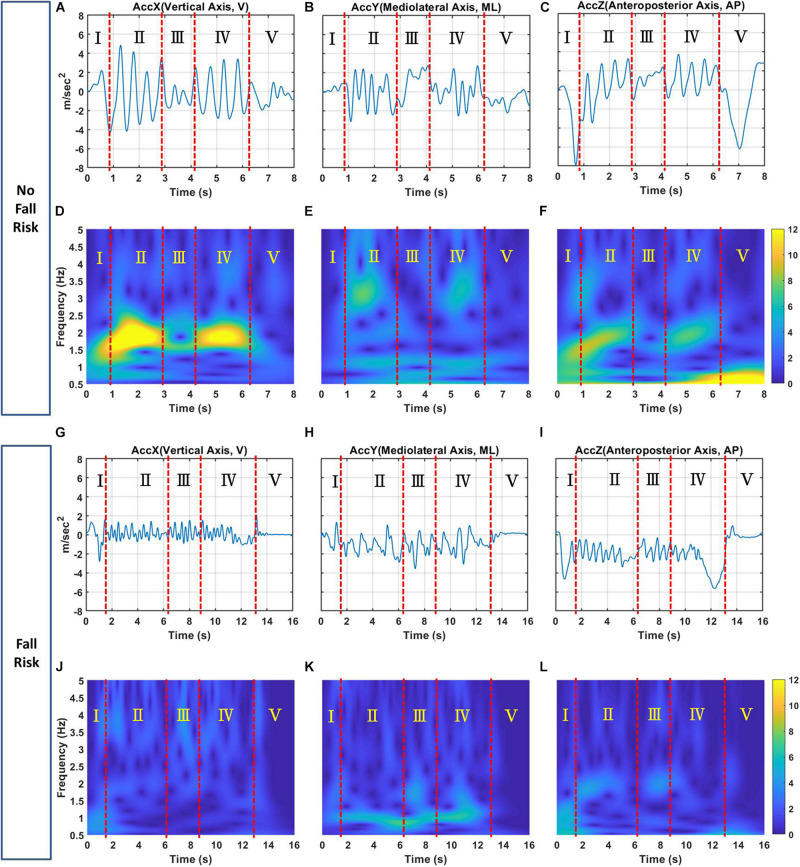
Examples of the **(A)** X-, **(B)** Y-, and **(C)** Z-axis acceleration signals for a subject with no fall risk; **(D–F)** corresponding TF images of triaxial acceleration signals, respectively. Examples of the **(G)** X-, **(H)** Y-, and **(I)** Z-axis acceleration signals for a subject with a fall risk; **(J–L)** corresponding TF images of triaxial acceleration signals, respectively. The X-, Y, and Z-axes correspond to the V, ML, and AP directions, respectively. Zones I, II, III, IV, and V represents the sit-to-stand, walk-F, turning, walk-B and stand-to-sit phases, respectively, of TUG. The color bar represents the magnitude of the TF energy.

Figure 4 shows examples of tri-axial acceleration signals in the time-domain for subjects with and without a fall risk and their corresponding TF images.

(1) For the X-axis, this axis is the vertical acceleration signal in time domain for the no-risk and at-risk subjects showed as Figures 4A,G, respectively. Figures 4A,G can be transformed through TFA to obtain TF images showed Figures 4D,J. Figure 4D clearly shows that the no-risk subject had two regions of interest in zones II and IV of the TF image corresponding to the walk-F and walk-B phases. The TF energy was 10–12, and the frequency was 1.5–2.5 Hz. Similarly, Figure 4J shows that the at-risk subject had regions of interest in zones II and IV corresponding to the walk-F and walk-B phases. The TF energy was 0.5–3, and the frequency was 1.5–2.5 Hz. Additionally, the turning phase showed obvious difference in Zone III between Figures 4D,J. The TF energy was 6–8, and the frequency was 1.5–2.0 Hz for no-risk subject. On the contrary, the TF energy was relatively low for no-risk subject. This is consistent with previous study (Drover et al., 2017; Wu et al., 2019), which noted that turn-based features are important predictors because they contain useful biomechanical information.

(2) For the Y-axis, this axis is the mediolateral acceleration signal in time domain for the no-risk and at-risk subjects showed as Figures 4B,H, respectively. Figures 4B,H can be transformed through TFA to obtain TF images showed Figures 4E,K. Figure 4E shows that the no-risk subject had two regions of interest in zones II and IV of the TF image corresponding to the walk-F and walk-B phases. The regions had high TF energies of 5–8 and 4–6, respectively, corresponding to frequencies of 2.5–3.5 and 1–1.3 Hz, respectively. Similarly, Figure 4K shows that the at-risk subject had regions of interest in zones II and IV corresponding to the walk-F and walk-B phases. Only one region had a high TF energy of 4–6 with a frequency of 1–1.3 Hz. In the Walk_F and Walk_B phases, the TF image showed the energy of mobility, which is supposedly related to the body and the arm swing when walking. Because of the walking duration, the arm swing is associated with postural stability (Meyns et al., 2013) can enhance gait stability (Bruijn et al., 2010).

(3) For the Z-axis, this axis is the anteroposterior acceleration signal in time domain for the no-risk and at-risk subjects showed as Figures 4C,I. Figures 4C,I can be transformed through TFA to obtain TF images showed Figures 4F,L. Figure 4F shows that the no-risk subject had two regions of interest in zones II and IV of the TF image corresponding to the walk-F and walk-B phases. The TF energy was 6–9, and the frequency was 1.5–2.5 Hz. Similarly, Figure 4L shows that the at-risk subject had regions of interest in zones II and IV corresponding to the walk-F and walk-B phases. The TF energy was 2.5–4, and the frequency was 1.5–2.5 Hz. The body will move forward to maintain balance while walking, and the AP-axis is seemly an important axis.

In summary, the no-risk subjects had higher TF energy than the at-risk subjects in zones II and IV corresponding to the walk-F and walk-B phases for all three axes. This is a reasonable assumption that no-risk subjects must have greater muscle strength or energy when walking than those with at-risk subjects. In addition, the no-risk subjects had obviously higher TF energy than the at-risk subjects did during the sit-to-stand (Zone I) and stand-to-sit (Zone V) phases in the Z-axis, referring to the transition subtask involving standing up and sitting down and these two abilities are largely related to strength and power of the lower extremities (Weiss et al., 2013). Moreover, the body must bend in forward–backward displacement. It is also reasonable to infer that the no-risk subjects had more energy to stand up or sit down than the at-risk subjects did. Regarding the differences located between 1 and 3 Hz approximately, the past literature (Schneider et al., 2010; Kline et al., 2016) have showed the frequency for movements along the longitudinal axis during running peaks at approximately 3 Hz, both in the activity and viewed movement conditions. They reported that a strong relationship exists between intrinsic and extrinsic oscillation patterns during exercise. A frequency of approximately 3 Hz seems to be dominant in different physiological systems (e.g., heart rate and brain cortical activity). Additionally, Robert C. et, al. mentioned that when the step frequency fell in the range of 0.5–3 Hz, the activity was identified as walking (Wagenaar et al., 2011). Compare with these results, we assume TF images may be used as an auxiliary tool to support medical professionals for clinically assessing fall risk.

#### Parameter Optimization for AE and Reconstruction

**FIGURE 5:**
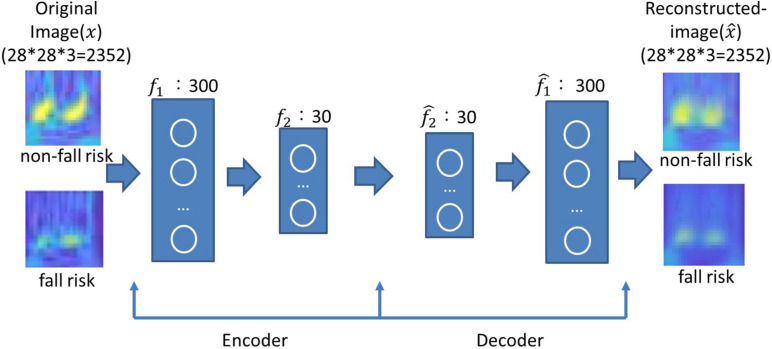
Examples of original and reconstructed images for subjects without and with a fall risk. A two-layer AE was used, where the encoder layers had 300–30 neurons and the decoder layer had 30–300 neurons.

The number of neurons was chosen according to the grid search strategy to minimize the mean squared error (Hinton and Salakhutdinov, 2006). The number of neurons in the first layer ranged from 100 to 500 in intervals of 100, and the number of neurons in the second layer ranged from 10 to 30 in intervals of 5. The mean squared error was obtained by averaging ten runs. As presented in Table 4, the minimum mean squared errors for the X-, Y- and Z-axes were 15.34, 12.03, and 9.73, respectively. These corresponded to 300 and 30 neurons in the first and second layers, respectively, for all three axes. Image reconstruction was carried out with 300–30 neurons for the encoder and 30–300 neurons for the decoder. As shown in Figure 5, the reconstructed image successfully restored the original image. Unsurprisingly, the latent features were useful for object recognition and other visual tasks (Ng, 2011).

**TABLE 4:** Mean squared errors for different combinations of neuron numbers in the first and second layers of the two-layer AE for the X-axis (V), Y-axis (ML), and Z-axis (AP).

**Axis**	**The neuron number of 1st layer**	**The neuron number of 2nd layer**				
		**10**	**15**	**20**	**25**	**30**
X-axis (V)	100	18.39	21.96	22.39	21.88	21.24
	200	23.07	23.52	19.93	19.77	22.06
	300	18.32	19.07	18.23	16.49	15.34*
	400	21.69	22.91	20.88	22.35	20.10
	500	21.62	20.58	18.65	20.65	18.52
Y-axis (ML)	100	16.13	14.02	15.01	13.47	15.11
	200	15.35	14.64	13.63	13.49	13.67
	300	14.11	13.59	13.43	12.46	12.03*
	400	14.15	13.01	12.69	12.43	13.09
	500	13.89	13.15	13.09	12.25	12.54
Z-axis (AP)	100	12.88	11.89	11.14	13.38	11.97
	200	15.37	14.05	10.63	14.14	10.47
	300	12.16	11.43	11.10	12.25	9.73*
	400	13.35	12.92	13.97	11.54	14.35
	500	14.23	12.66	12.21	11.21	10.30

*The minimum of mean square error.

#### Analysis of the Stacked Autoencoder

**FIGURE 6:**
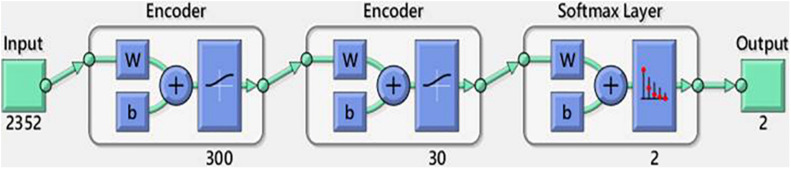
Diagram of the SAE.

As shown in Figure 6, the SAE had an input of 2,352 pixels with an encoder layer of 300–30 neurons and a softmax classifier layer with two classes. Table 5 presents the classification results of the SAE. The classification accuracies were 89.1, 93.4, and 94.1% along the X-, Y-, and Z-axes, respectively. The sensitivities were 85.5, 94.1, and 94.6%, respectively. The specificities were 92.7, 92.7, and 93.6%, respectively. The SAE performed better along the Y- and Z-axes than along the X-axis. Thus, the latent features of the Y- and Z-axes may offer more predictive ability for DNN-based evaluation.

**TABLE 5:** Classification results with the SAE.

**Axis**	**Accuracy**	**Sensitivity**	**Specificity**
X-axis(V)	89.1 ± 1.0%	85.5 ± 4.2%	92.7 ± 3.2%
Y-axis(ML)	93.4 ± 1.7%	94.1 ± 2.2%	92.7 ± 3.8%
Z-axis(AP)	94.1 ± 1.6%	94.6 ± 2.9%	93.6 ± 2.4%

*The accuracy, sensitivity, and specificity are average ± SD values for ten runs.*

Tables 3, 5 indicate that the DNN-based evaluation performed much better than the feature-based evaluation. Thus, it is a viable approach for fall detection. In addition, the Y- and Z-axes are both important for classification. With regard to the Y-axis, swinging arms are associated with postural stability and can enhance gait stability (Wu et al., 2019) and mobility function. With regard to the Z-axis, this is important to transitions involving standing up or sitting down, where the body must bend in forward–backward displacement. These results are similar to those of previous study (Wu et al., 2019), who identified features extracted along the Z-axis for TUG tasks as significant and Z-axis is seemly an important axis.

## Conclusion

In this paper, tri-axial accelerometer data were collected from a cheap wearable sensor, and TFA was used to convert the data into TFRs. These TF images offered abundant and discriminative information such as time, frequency and spectral energy-related power in five phases of TUG, which clarified specific TUG aspects or subtasks were impaired in mobility. High energy-related power of no- risk subjects in both walk phases (walk-F and walk-B) and transition phases (sit-to-stand and stand-to-sit) phases can be observed obviously from TF images for all three axes and AP axis, respectively. We also applied SAE model, DNN-based evaluation, to classify TFRs of elderly subjects for assessing the mobility and fall risk. Experimental results show that the DNN-based evaluation offers much considerably accuracy, sensitivity and specificity rates. Moreover, the results indicated the superior performance of DNN-based evaluation over feature-based evaluation. Further, the discrimination analysis of Y and Z axes seems to be more important than that of X axis.

In the future, we will continuously work on DNN-based evaluation of fall risk for the elderly. This innovative method based on the artificial intelligence technology, i.e., DNN-based evaluation, can be widely used in wearable sensing technology, smart home development and continuous monitoring technologies for real-time measurement and recording of various physiological signals. We trust it will improve the accessibility and convenience of people’s medical care.

## Data Availability Statement

The data analyzed in this study is subject to the following licenses/restrictions: we have signed a confidentiality agreement with the hospital. Requests to access these datasets should be directed to C-HL, sweat0430@mail.ntust.edu.tw.

## Ethics Statement

The studies involving human participants were reviewed and approved by Tsaotun Psychiatric Center, Ministry of Health and Welfare (IRB No. 104013). The patients/participants provided their written informed consent to participate in this study.

## Author Contributions

S-HC, C-HL, BJ, and T-LS: conceptualization and validation. C-HL, S-HC, and T-LS: data curation. S-HC: formal analysis. C-HL, BJ, and T-LS: investigation. S-HC and C-HL: methodology, software, and writing – original draft. BJ and T-LS: resources and writing – review and editing. All authors contributed to the article and approved the submitted version.

## Conflict of Interest

The authors declare that the research was conducted in the absence of any commercial or financial relationships that could be construed as a potential conflict of interest.
